# Can the left hand benefit from being right? The influence of body side on perceived grasping ability

**DOI:** 10.3758/s13414-024-02983-7

**Published:** 2024-11-18

**Authors:** Rachael L. Taylor, Neil McLatchie, Sally A. Linkenauger

**Affiliations:** 1 School of Psychology, Lancaster University, Lancaster, UK; 2 School of Psychology, University of Kent, Canterbury, CT2 7NP UK

**Keywords:** Right-handed individuals, Grasp ability, Virtual reality, Mirror visual feedback

## Abstract

Right-handed individuals (RHIs) demonstrate perceptual biases towards their right hand, estimating it to be larger and longer than their left. In addition, RHIs estimate that they can grasp larger objects with their right hand than their left. This study investigated whether visual information specifying handedness enhances biases in RHIs’ perceptions of their action capabilities. Twenty-two participants were placed in an immersive virtual environment in which self-animated, virtual hands were either presented congruently to their physical hand or mirrored. Following a calibration task, participants estimated their maximum grasp size by adjusting the size of a virtual block until it reached the largest size they thought they could grasp. The results showed that, consistent with research outside of virtual reality, RHIs gave larger estimates of maximum grasp when using their right physical hand than their left. However, this difference remained regardless of how the hand was virtually presented. This finding suggests that proprioceptive feedback may be more important than visual feedback when estimating maximum grasp. In addition, visual feedback on handedness does not appear to enhance biases in perceptions of maximum grasp with the right hand. Considerations for further research into the embodiment of mirrored virtual limbs are discussed.

**Supplementary information:**

The online version contains supplementary material available at 10.3758/s13414-024-02983-7.

## Introduction

Right-handed individuals (RHIs) demonstrate a consistent preference for their right hand in most unimanual tasks, even in instances where using their left hand would be more biomechanically efficient (Bryden et al., 2004). For example, RHIs will opt to use their right hand more often than their left even when reaching into the left side of space (Gonzalez et al., 2007). Moreover, RHIs show a strong right-hand preference for picking up various objects such as geometric three-dimensional (3D) shapes (Gabbard et al., 2003), tools (Mamolo et al., 2004, 2006) and blocks (Gonzalez et al., 2007; Netelenbos & Gonzalez, 2015; Stone et al., 2013). Various factors may underlie this strong right-hand preference in RHIs, including differences in cortical representation of the right hand, subsequent perceptual biases that favour the right side of the body, and processing of sensory feedback from moving the right arm.

Various studies provide evidence that RHIs have asymmetries in the cortical representation of their left and right hands. For example, Jung et al. (2003) reported enlarged hand representation in the left somatosensory cortex compared to the right somatosensory cortex in RHIs. Furthermore, RHIs demonstrate greater cortical representation of the right hand in the left motor cortex compared to the left hand in right motor cortex (Amunts et al., 1996; Volkmann et al., 1998), and the threshold for muscle activation in the right arm is lower than that for the left, indicating greater excitability of the left motor cortex (Triggs et al., 1999). Left-handed individuals (LHIs), however, do not exhibit similar asymmetries in the representation of their left hand (Amunts et al., 1996; Sörös et al., 1999).

These cortical asymmetries correlate to RHIs’ perceptions of the size of their left and right hands (Linkenauger et al., 2009). Linkenauger et al. asked both RHIs and LHIs to estimate the size and length of their hands and arms by visually matching them to the length of a tape measure. RHIs underestimated the length and size of their left arm and hand whilst the right was perceived accurately. LHIs estimated both of their arms and hands to be the same size and length, suggesting that the perception that the right hand is larger in RHIs is rooted in the enlarged sensorimotor representation of the right hand (Coelho et al., 2016; Linkenauger et al., 2009). If RHIs perceive their right hand as being visually larger, it may follow that RHIs also estimate their capabilities with their right hand to be greater than their left for actions where hand size is relevant.

How we perceive our opportunities for action (also known as ‘affordances’; Gibson, 1979) depends on how our morphology limits the extent to which an action can be performed, with the maximum extent known as an ‘action boundary’ (Fajen, 2005). While our actual morphology is important, what may also be important is our perception of our morphology. Indeed, Linkenauger et al. (2009) explored RHIs’ estimates of their action boundaries with each hand for reaching and grasping. By measuring perceived graspability as a ratio of perceived maximum grasp and actual maximum grasp, they found that RHIs overestimated the maximum size of an object they could grasp more with their right hand than their left. This is despite no real differences in grasping ability between the hands. As with perceptions of hand size, LHIs did not demonstrate any asymmetry in the perception of maximum grasp with either arm. Linkenauger et al. argue that the exaggerated perception of the right hand in RHIs is adaptive in that it encourages greater use of the right hand than the left. Therefore, the strong right-hand preference RHIs exhibit may be attributed to the perception that the right hand is visually larger than the left and thus has greater capabilities.

Another factor that promotes greater use of the right hand in RHIs may lie in better processing of somatosensory feedback from moving the right hand (Flowers, 1975). Flowers suggests that differences in skill between the preferred and non-preferred hand is primarily due to a more efficient sensorimotor feedback loop in which sensory feedback is transmitted back to the motor system to correct movement. This theory has been extended to focus specifically on processing of visual feedback from movement. That is, the left hemisphere may be better able to process visual feedback obtained from visually guided actions performed with the right hand (Flindall et al., 2013, 2015). However, it is important to note that the sensory feedback referred to by Flowers (1975) is not just limited to vision, but instead refers to sensory feedback from any source such as proprioception (Carson et al., 1990). Some evidence suggests that the right-hand advantage in actions, such as reach-to-point, persists even without vision (Roy et al., 1994). Therefore, it is questionable whether visual feedback plays a role in the asymmetry in performance between the left and right hands in RHIs.

A key question here, then, is whether the biases in perception of RHIs’ action capabilities are enhanced by visual factors. Linkenauger et al. (2009) find that the perception that the right hand and arm are larger in RHIs coincides with greater estimations of reach and grasp. This perception of having greater action capabilities may be reinforced by more efficient visuomotor feedback during visually guided actions that allows the right hand to perform more skilled movements. However, biases in the perception of hand size arguably originate from cortical asymmetries in hand representation. Similarly, the right hand’s greater skill in various actions may also be rooted in hemispheric asymmetries in which sensory feedback aside from vision from the right arm is processed more efficiently. It is possible, therefore, that the relationship between the perception of larger right-hand size and greater action capabilities is not that larger right-hand size leads to estimates of greater capability. Rather, greater capabilities with the right hand lead to the perception that the right hand is larger in RHIs. If this is the case, visual feedback on the hand being used would not sufficiently explain differences in estimates of RHIs’ action boundaries between the left and the right hands. Thus, the main question is whether visual feedback on handedness alone is sufficient to alter one’s perceptions of their action capabilities.

Previous research exploring perceptions of maximum grasp has found that manipulating the visual feedback associated with moving the hands can change people’s perceptions of their abilities. For example, magnifying the real hand to increase its visual size can lead to the perception that larger objects can be grasped (Linkenauger et al., 2011). In addition, Readman et al. (2021) found that calibrating people to a large virtual hand led to larger estimates of maximum grasp than when they were calibrated to a small virtual hand. Thus, manipulating the visual sizes of the hands using virtual reality (VR) can elicit changes in action perception. One key question is whether manipulating visual feedback specifying the hand being used can exploit pre-existing biases in hand size, rather than actively changing hand size in a dramatic manner.

To explore whether visual feedback of handedness enhances right-handed biases in action perception, we used VR to isolate visual feedback from other types of sensory feedback that would normally be experienced when moving the hand. We placed RHIs in a virtual environment (VE) with hand-tracking sensors and mirrored the hands, so that when the participant’s left hand moved an animation of the right hand would be shown in the virtual environment. We then took participants’ estimates of their maximum grip size by adjusting the length of a virtual white block until it reached the perceived maximum size that they could grasp. We have two key hypotheses. First, RHIs perceive their action capabilities to be greater when using their right hand than their left. Thus, we predict that participants will give significantly larger estimates of grip when estimating with their right hand than with their left, in line with previous research conducted outside of VR. Our second hypothesis is that the visual bias that the right hand is larger than the left underlies the perception that the right hand is more capable than the left. Therefore, we predict that when the hand is animated as the right in the virtual environment, estimates of maximum grip will be larger than when the hand is animated as the left in the virtual environment.

## Methods

### Transparency and openness

The study design, hypotheses and analysis plan were not preregistered. We report how we determined our sample size, all data exclusions (if any), all manipulations and all measures in the study. Data were analysed using SPSS version 28.0. Analysis code is not available as SPSS does not have the functionality to export syntax. Data and study materials are publicly available on the Open Science Framework at: https://osf.io/nsg8r/.

### Participants

We recruited 22 right-handed (16 female, four male, two prefer not to say) and three mixed-handed (two female, one male) individuals from Lancaster University (age range: 18–21 years, *M* = 18.84, *SD* = 1.4). This sample size was chosen because Linkenauger et al. (2009) used a sample of 15 right-handed participants in a similar grasping estimate study and found a strong effect of hand (*η*_*p*_^*2*^ = 0.25). However, due to technical issues with the head-mounted display, three participants could not complete the full study.

Therefore, the final sample consisted of 19 right-handed (14 female, three male, two prefer not to say) and three mixed-handed (two female, one male, with laterality scores ranging from + 20 to + 58.35) individuals from Lancaster University (age range: 18–21 years, *M* = 18.68, *SD* = 0.95). Sensitivity power analysis using MorePower 6.0.4 (Campbell & Thompson, 2012) with the final sample of 22 was conducted, which shows that a repeated-measures ANOVA with three factors would be sensitive enough to detect an effect size of *η*_*p*_^*2*^ = 0.29 (α = 0.05, power = 0.8).

Handedness was assessed using the 10-item Edinburgh Handedness Inventory (Oldfield, 1971), with scores ≥  + 60 being assigned as right-handed, scores between + 59 to -59 being assigned as mixed-handed, and scores ≤ -60 being assigned as left-handed. These cut-off points were based on research by Milenkovic and Dragovic (2012) and Veale (2013). The mixed-handed individuals were recruited as they all stated a strong right-hand preference for writing, with writing being a key predictor of handedness (Bryden, 1977; Rigal, 1992). Participants were recruited from Lancaster University SONA system and were awarded 2 credits as part of their undergraduate psychology course. All participants first gave written informed consent before participating. Data were collected in February 2022.

### Materials

For descriptions of specific objects in the virtual environment, it is difficult to give exact real-world dimensions as Unity Version 2021.1.24f1 (Unity Technologies, San Fransisco, CA, USA) uses unique scaling. However, it is a standard assumption in Unity that 1 unit in Unity is equal to 1 real-world metre (Unity Technologies, 2018). We convert units in Unity to their equivalent real-world dimensions for ease of understanding.

### Virtual environment

**Fig. 1:**
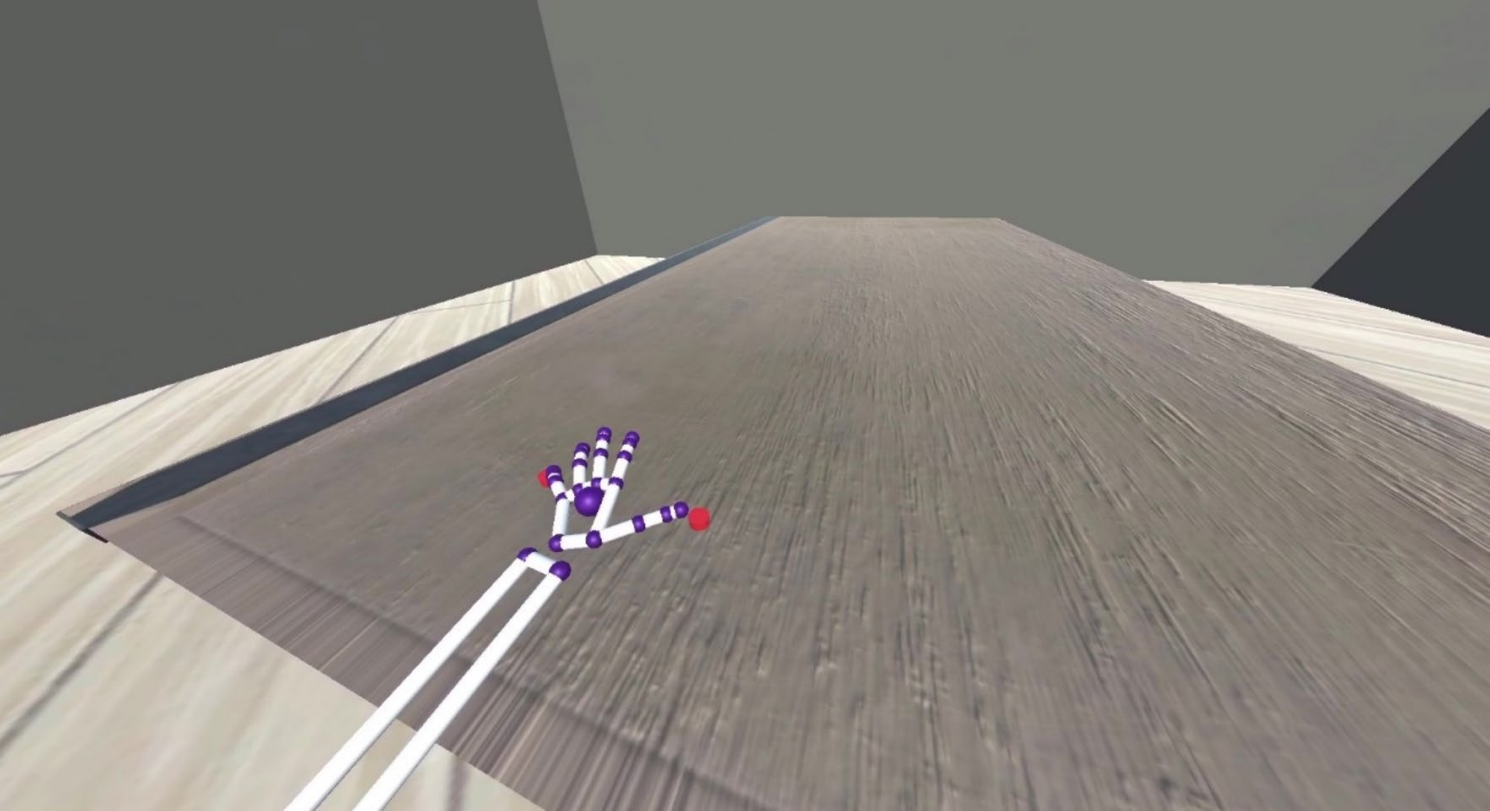
One of the calibration trials, as shown when using the left virtual hand

Participants wore an Oculus CV1 VR headset which placed participants in a VE that consisted of a wooden Table (70 cm height, 3 m length) in a plain room with three grey walls and a light wooden floor. During the calibration phase, a pair of small red circles called ‘calibration dots’ (2 cm in diameter, 2 cm in depth) were placed on top of the virtual table to the left, right or centre of the participant. In the estimation phase, a white block (6 cm, 8 cm, 10 cm, 12 cm, 14 cm or 16 cm in length, 5 cm in length and 5 cm in depth) was placed 20 cm away from the participant on the virtual table. Eye height was standardised for all participants at 46 cm from the virtual table, with participants placed 8 cm away from the virtual table. A LEAP motion sensor was attached to the front of the headset that tracked participants’ hand movements in real time. Hand movements were animated using Leap Motion ‘capsule hand’ models (Leap Motion, Inc., San Francisco, CA, USA). These hand models are white with small, coloured spheres indicating the position of different joints in the hand and wrist (see Fig. 1). The capsule hands were used because more realistic hand models we had tested became distorted when we attempted to mirror them. Therefore, these simplistic hands were the best option to ensure participants could accurately move the virtual hand. The virtual hands are based on the size of the user’s real hands in front of the LEAP motion sensor (Ultraleap, n.d.), so any individual differences in hand size should not affect calibration.

### Design

This was a 2 × 2 × 6 within-subjects design. There were three factors: first was the physical hand used during the calibration (right vs. left); second was the virtual hand presented during the calibration (right vs. left); third was the starting size of the white block during the estimation trials. We varied starting size to control for hysteresis (6 cm, 8 cm, 10 cm, 12 cm, 14 cm or 16 cm). Hysteresis is a phenomenon in which what is perceived depends on recent sensory experiences and can therefore affect future judgements (You et al., 2011). By varying starting size, we ensure that participants are completing the task properly and not simply repeating the same estimates over and over again, as we would expect that larger block sizes would elicit larger estimations of grasp based on hysteresis. The dependent measure was the maximum estimated grip, as measured by the final length of the white block after adjustment.

### Procedure

This study received ethical approval from the Faculty of Science and Technology ethics committee at Lancaster University. Before being placed in the virtual environment for each condition, participants were told which physical hand to use and whether the visual feedback would be normal or mirrored. Then, participants took part in two phases of the experiment for each condition: the calibration and the estimation phases. Please note that for each condition, the participant performed the estimation task immediately after calibrating.

### Calibration

Participants first performed a calibration task, which was designed for them to gain experience in how movement of their real hands mapped onto movement of the virtual hands. This perceptual-motor coupling was vital for participants to make more informed estimates of their action capabilities using the virtual hands. Participants sat at a table with their chair pushed back against the wall so that they would not accidentally hit the table during the calibration. During the calibration, participants were instructed to stretch their hand out so that the thumb and little finger of the virtual hand were each touching one of two red calibration dots positioned on the virtual table (see Fig. 1) and to follow the calibration dots as they changed position. The positions of the calibration dots were presented in a randomised order (see Appendix in the Online Supplemental Material for exact positions of the calibration dots). When the right virtual hand was used, the calibration dots were situated from the right to the centre of the participant. When the left virtual hand was used, the calibration dots were situated from the centre to the left of the participant. This was so that participants did not need to reach across their body and strain to reach the calibration dots. The calibration lasted for 60 trials per condition, taking between 5 and 7 min, with the mirrored calibration trials often taking longer than the non-mirrored trials due to the visuomotor adjustment needed to control the virtual hand. We opted for 60 calibration trials because previous studies using similar methodologies in virtual reality have used similar numbers of calibration trials (often fewer) and found strong effects relating to the adaptation to the abilities of the virtual hand. For example, Lin et al. (2020) employed 54 calibration trials, while Readman et al. (2021) used 30.

### Estimation

**Fig. 2:**
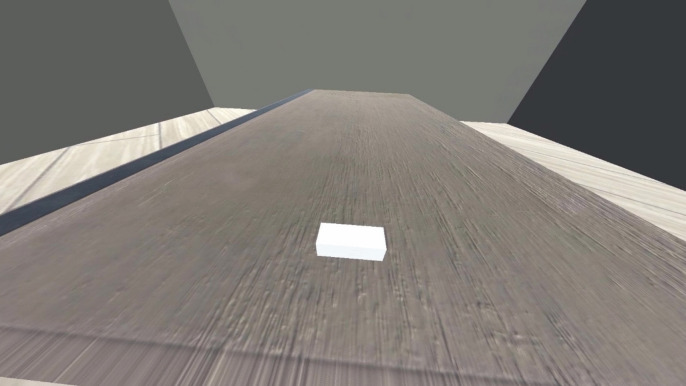
One of the white blocks used in the estimation trials

After the calibration, participants were instructed to keep their hand in their lap so that they no longer received visual feedback from the virtual hand nor proprioceptive feedback from their real hand during the estimation phase. Then, the white block appeared on the table in front of the participant (see Fig. 2). The researcher increased or decreased the white block’s length by 1 mm – using the left and right arrow keys on the computer – until the participant verbally stated that the length of the white block was at the maximum width that they could grip from the top using the thumb and index finger of the actual hand they had been moving throughout the calibration phase. Participants could also state that the block was fine as is. The final size of the white block after adjustment was saved after each trial. The estimation phase lasted 12 trials, with two trials per starting length presented in a randomised order. This procedure was repeated four times, once for each condition. Overall, the study (with all four conditions) took around 30 min to complete.

## Results

We conducted repeated-measure analyses of variance (ANOVAs) on the final sample of 22 to test our key hypotheses. For all one degree-of-freedom tests, we report both p values and Bayes factors. Bayes factors are a continuous measure of relative evidence, calculated as the ratio of the probability of the data under one hypothesis (e.g., the experimental hypothesis (H1)) to the probability of the data under another hypothesis (e.g., the null hypothesis (H0)). We used the Dienes and McLatchie (2018) R script calculator to calculate Bayes factors, and we provide robustness regions (RRs) to indicate the range of effect sizes that support our conclusions. For more information on robustness regions, please see McLatchie et al. (2020).

We interpret Bayes factors greater than 3 and 10 as moderate and strong evidence, respectively, in support of the experimental hypothesis, and Bayes factors less than 0.33 and 0.10 as moderate and strong evidence, respectively, in support of the null hypothesis. Bayes factors between 0.33 and 3 are considered to provide weak or inconclusive evidence. Please note that these thresholds are provided to aid interpretation and transparency in decision-making, but the Bayes factor itself is a continuous measure. For further discussion and comparison of Bayes factors and p values, see Lakens et al. (2018).

**Fig. 3:**
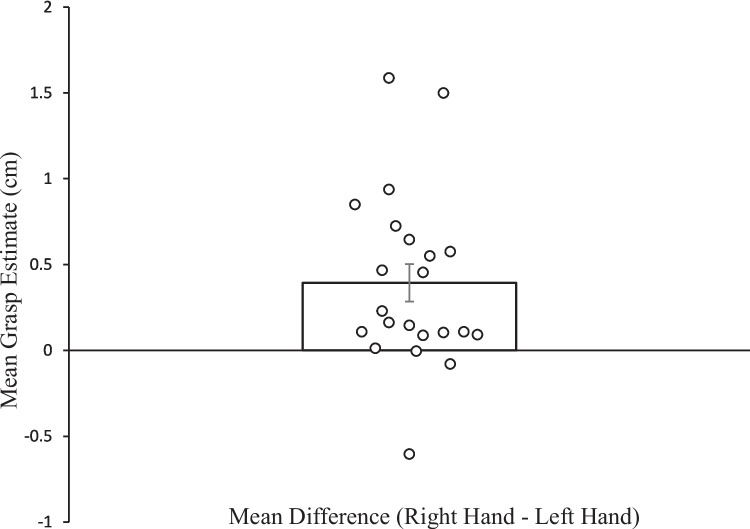
Mean difference in grasping estimates between the left and right hand. The error bar represents standard error. Circles represent the mean difference in grasping estimates for each participant

**Fig. 4:**
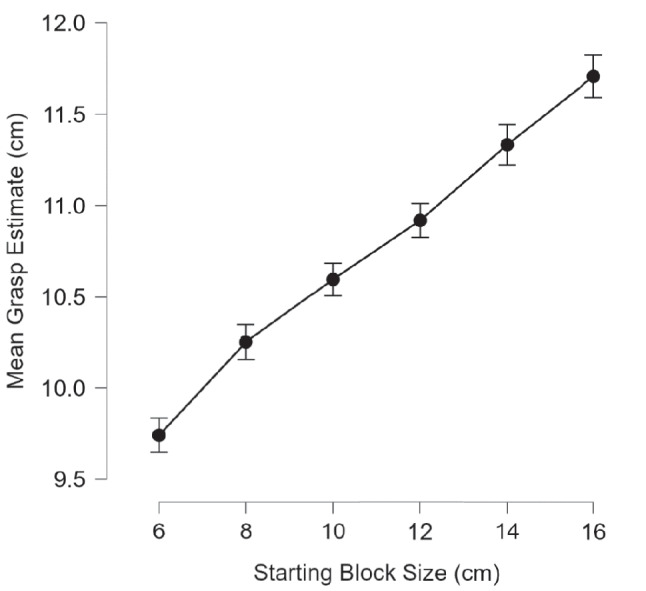
Mean grasp estimates as a function of block starting size. Error bars represent standard error

A within-subjects ANOVA was conducted with actual hand (right vs. left), virtual hand presented (right vs. left) and white block starting length (6 cm, 8 cm, 10 cm, 12 cm, 14 cm or 16 cm) as the independent factors and *maximum* grip estimate (as measured by the length of the block after adjustment) as the dependent factor. There was a main effect of actual hand used, *F*(1, 21) = 13.02, *p* = 0.002, *η*_*p*_^*2*^ = 0.38, *B*_*H(0,2.64)*_ = 54.05*, RR[0.04,47.30*^1^*],* with participants giving significantly larger estimates of maximum grasp with their right hand (*M* = 10.95 cm, *SD* = 1.7 cm) than their left hand (*M* = 10.56 cm, *SD* = 1.63 cm; see Fig. 3) and Bayes factors providing strong support for H1. There was also a main effect of starting length, *F*(2.127, 44.67) = 37.61, *p* < 0.001, *η*_*p*_^*2*^ = 0.64, with mean estimates of grasp increasing as starting length increased (see Fig. 4). However, there was no main effect of virtual hand presented *F*(1, 21) = 0.001, *p* = 0.98, *η*_*p*_^*2*^ = 0.00005,* B*_*N(0,0.39)*_ = 0.19*, RR[0.28,∞*^2^*]* with no significant differences in estimates of maximum grasp between the virtual right hand (*M* = 10.76 cm, *SD* = 1.7 cm) and the virtual left hand (*M* = 10.76 cm, *SD* = 1.66 cm), with the Bayes factor indicating moderate evidence for H0. There were no significant interactions between the real hand and virtual hand (*F*(1, 21) = 0.48, *p* = 0.49, *η*_*p*_^*2*^ = 0.02), *B*_*N(0,0.39)*_ = 1.20*, RR[0,1.61],* the real hand and block starting length (*F*(5, 105) = 0.54, *p* = 0.75, *η*_*p*_^*2*^ = 0.03), the virtual hand and block starting length (*F*(3.083, 64.73) = 1.16, *p* = 0.52, *η*_*p*_^*2*^ = 0.05) nor real hand, virtual hand and block starting length (*F*(5, 105) = 0.81, *p* = 0.55, *η*_*p*_^*2*^ = 0.04).

## Discussion

Here, we investigated perceptions of grasping ability in a virtual environment, exploring how manipulating visual feedback specifying hand use would impact these perceptions. Firstly, we predicted that participants would give larger estimates of maximum grasp when using their right hand than their left. Secondly, we predicted that, if visual feedback on handedness significantly influences RHIs’ perceptions of their action capabilities, estimates of maximum grip will be larger when the hand is animated as the right in the virtual environment.

Our results support the first hypothesis, as mean estimates of maximum grip size were significantly larger in the right-hand conditions than in the left-hand conditions, and Bayes factors provide very strong support for H1. This finding replicates similar research conducted outside of VR (Linkenauger et al., 2009). However, we found no evidence that the visual presentation of the hand impacts maximum grip estimates, as we found was no evidence for a significant difference between estimates made after right virtual hand presentation or left virtual hand presentation, and with Bayes factors providing moderate evidence for H0. Finally, we found that as the size of the white block increased, mean maximum estimates of grip size did indeed increase, which is indicative of hysteresis. That is, participants’ judgements of maximum grip were impacted by the initial presentation of block size.

Previous research demonstrates that the perceived action boundary for grasping can be manipulated through adjusting visual information on hand size (Linkenauger et al., 2011; Readman et al., 2021). Thus, it is possible for people to adjust their perceptions of their action boundaries after visually manipulating the relevant body part for the action. However, manipulating the visually specified hand during the calibration did not lead to any adjustment of perceptions of maximum grasp ability in this study. Since participants estimated that they could grasp significantly larger objects using their right hand in a similar manner to real-world studies (see Linkenauger et al., 2009), this suggests that participants may have primarily used proprioceptive feedback specifying the hand being used during the calibration when estimating their maximum grasp ability, rather than offline visual models of the hands.

One reason for this could be that estimating one’s action capabilities may involve the use of internal motor simulation (Witt & Profitt, 2008). Motor simulation theory (MST) suggests that all action involves a covert stage before motor execution (ME) (Jeannerod, 2001). This covert stage shares the same mechanisms as actual action, with neural activation in motor imagery (MI) overlapping with that seen in an executed action (O’Shea & Moran, 2017). A wide range of neurophysiological evidence supports this view, with MI and ME sharing brain regions such as pre-motor cortex, primary motor cortex, and supplementary motor area, although neural activation is generally weaker during MI (see O’Shea & Moran, 2017, for a review). As discussed in the *Introduction*, cortical asymmetries between the left and right hemispheres contribute to the perceptual biases towards the right side of the body in RHIs (Amunts et al., 1996; Volkmann et al., 1998). Thus, if participants were simulating the action when estimating their maximum grasp ability using their right hand, it is likely that they were employing some of the brain regions that underlie the right-hand perceptual bias in RHIs. Similarly, when estimating using their left hand, the lower degree of cortical representation in these motor areas would also play a role in their estimates. Therefore, the mirrored visual feedback experienced during the calibration would not alter estimations of maximum grip because during the estimation task there is a reliance on cortical structures involved in motor imagery rather than visual feedback.

An alternative explanation as to why visually manipulating the seen hand during the calibration did not significantly impact estimates of maximum grasp is that the effect of real hand found in previous research (Linkenauger et al., 2009) cannot be accounted for by the visual perception of the hands. That is, RHIs perceiving their right hand as larger does not in turn lead to estimates of greater grasping ability. Rather, we hypothesise that a perception of greater capabilities with the right hand drives the visual bias that the right hand is larger. Since the left hand does not possess the same level of skill and dexterity as the right in RHIs, this discrepancy in skill would lead to lower estimates of one’s action capabilities regardless of whether it took on the visual appearance of the ‘larger’ right hand. Moreover, since the visual perception that the right hand is larger may also be a consequence of enlarged sensorimotor representation in the left hemisphere (Amunts et al., 1996; Volkmann et al., 1998), it is possible that participants did not retain the visual perception that the right hand was larger when the left hand was controlling it. Thus, we tentatively suggest for the right hand to be perceived as visually larger it must be paired with both the greater skill and enlarged somatosensory representation of the physical right hand. A further point of discussion is whether the larger cortical representation of the right hand is also related to higher tactile acuity. Previous research has shown that sensory thresholds tend to be lower for the left hand in RHIs (Meador et al., 1998), but that effects of handedness may be task-specific (Duncan & Boynton, 2007; Summers & Lederman, 1990). This topic is beyond the scope of our paper, but is an interesting avenue for further research.

It is important to note that people’s perceptions of their hands are reliably distorted beyond the effect of handedness discussed here. Implicit hand maps, generated from haptic information, are systematically distorted, with finger length being underestimated while hand width is overestimated (Longo & Haggard, 2010). Interestingly, however, conscious perception of hand shape is more veridical (Longo & Haggard, 2010). Longo and Haggard argue that implicit hand maps may be corrected with supplemental information from vision and efferent copies of motor commands when performing action. Thus, these implicit hand map distortions differ from the perceptual biases shown by RHIs, which are evident in explicit judgements of hand size with full vision of the hands available (Linkenauger et al., 2009).

In addition, while people use intrinsic information about their bodies to judge their affordances (Mark, 1987; Warren & Wang, 1987), they can use this in combination with visual, haptic and proprioceptive feedback received through everyday experiences (Adolph & Hoch, 2019) or through performing a target action relevant to the affordance (Franchak et al., 2010). In our calibration task, participants would receive rich visual and proprioceptive feedback informing them of their potential grasping ability. In this case, we do not expect that implicit distortions of hand shape and size would affect explicit affordance judgements in our task. However, whether these implicit distortions may affect other affordance judgements is an interesting question that could be explored further.

Whether participants embodied the mirrored virtual hands well enough to consider them in their estimates is an important question. Research exploring illusions of body ownership over virtual limbs highlights the importance of congruent cross-modal multisensory stimulation between the real and virtual body (Kokkinara & Slater, 2014; Sanchez-Vives et al., 2010; Slater et al., 2008). The visuo-proprioceptive mismatch during the mirrored calibration could disrupt embodiment of the virtual limb, preventing offline models of hand representation from changing in response to the altered visual feedback experienced during the calibration. However, recent research has found that embodiment of mirrored virtual hands can be achieved in healthy users, with participants having both a sense of agency and ownership over the mirrored virtual hands (Heinrich et al., 2020). While more realistic avatars can create a stronger sense of embodiment (Ogawa et al., 2019), Heinrich et al. found that the use of the unrealistic capsule hands (the same models used in our study) did not prevent a sense of embodiment from being achieved. While Heinrich et al. (2020) provide some evidence of embodiment of mirrored virtual hands, the literature is currently limited and therefore more research using different measures of embodiment is necessary.

Overall, this study provides insight into the nature of the perceptual biases that RHIs experience towards the right side of the body. Estimates of maximum grasp ability were dependent on the actual hand being used and not the visual information specifying the hand being used. This suggests that underlying cortical and sensory representations of the right hand are what primarily underlie the perceptual biases towards the right side of the body, and that visual information about the lateralisation of the hand may not enhance this bias. Additionally, proprioceptive information on the hand being used appears to be the most important when estimating maximum grasp. However, it is possible that the effect of virtual hand size was too small to be detected by our current sample, as the sensitivity analysis suggested that we would have good power to detect a large effect size. Though the lack of significant effect of virtual hand is not believed to be down to a lack of embodiment, more research should be conducted to explore the embodiment of mirrored virtual limbs.

### Constraints on generality

As all participants in this study were healthy undergraduate university students aged between 18 and 21 years, it is possible that the findings would not generalise to differently aged populations, such as older adults. This is because old age is associated with reduced mobility of the hand (Holt et al., 2013), hence reduced mobility could impact estimates of maximum grasp. Additionally, all were right-handed (or mixed-handed with right-hand writing preference), and so left-handed individuals may respond differently to the manipulations in this study, since they do not experience the same perceptual biases towards the left side of their body (Linkenauger et al., 2009). The experimental stimuli are all presented in a virtual environment; however, manipulating sensory feedback in the way we have done in this study is not easily achieved in the real world. Overall, the results of this study would best generalise to young, healthy, right-handed adults who complete the procedure within an immersive virtual environment.

## Supplementary information

Below is the link to the electronic supplementary material.

Supplementary file1 (DOCX 15 KB)

## Data Availability

The data used in analysis are publicly available on the Open Science Framework at: https://osf.io/nsg8r/.

## Footnotes

1 H1 was specified assuming that a difference of 50% in perceived grasp would constitute an upper limit in terms of an approximate effect size. As the left hand was estimated by participants to be 10.56 cm, this leads to an upper limit expected effect size of 5.28. Following Dienes (2011) we assumed a half normal prior distribution with a SD of 5.28 / 2 = 2.64.

2 H1 was specified using the obtained mean difference from the actual hand data, 10.95—10.56 = 0.39, to provide an approximate scale of effect for the main effect of hand and for the interaction between the real hand and virtual hand. Following Dienes (2011), we assumed a normal prior with mode = 0.39 and SD = 0.195.

## Code availability

The Unity build used for this experiment can be obtained by contacting the corresponding author.
