# Supplementary material for: Can the left hand benefit from being right? The influence of body side on perceived grasping ability
Source: Atten Percept Psychophys. 2024 Nov 18;86(8):2834–43. doi: 10.3758/s13414-024-02983-7 (PMC11652622; doi:10.3758/s13414-024-02983-7)
Supplement: Supplementary file 1 — Supplementary file1 (DOCX 15 KB) [file 13414_2024_2983_MOESM1_ESM.docx]

**Appendix: Coordinates of Calibration Circles**

| Calibration circle 1 (x-axis) | Calibration circle 2 (x-axis) | Both calibration circles (z-axis) |
| --- | --- | --- |
| .05 | .22 | .1 |
| .05 | .1 | .1 |
| .05 | .12 | .2 |
| .05 | .1 | .2 |
| .0625 | .175 | .1 |
| .0625 | .175 | .2 |
| .075 | .25 | .1 |
| .075 | .15 | .2 |
| .0875 | .225 | .1 |
| .0875 | .125 | .2 |
| .1 | .28 | .1 |
| .1 | .2 | .2 |
| .1 | .28 | .2 |
| .125 | .275 | .1 |
| .125 | .275 | .2 |
| .15 | .25 | .1 |
| .15 | .25 | .2 |
| .175 | .225 | .1 |
| .175 | .225 | .2 |
| .2 | .38 | .1 |
| .2 | .3 | .1 |
| .2 | .3 | .1 |
| .2 | .3 | .2 |
| .2 | .38 | .2 |
| .225 | .375 | .1 |
| .225 | .375 | .2 |
| .25 | .35 | .1 |
| .25 | .35 | .2 |
| .275 | .325 | .1 |
| .275 | .325 | .2 |

*Note.* The y-axis was kept constant at .47 across all trials. For calibration trials to the left side of space, simply make the x-axis values negative.
